# A Memoir on the Yellow Fever of the West Indies, *as It Occurred in the Year* 1838, *at St. Pierre, Island of Martinique*

**Published:** 1840-02-22

**Authors:** E. Rufz

**Affiliations:** Adjunct Professor at the School of Medicine of Paris


### MEDICAL EXAMINER.

DEVOTED TO MEDICINE, SURGERY, AND THE COLLATERAL SCIENCES.

No. 8.] PHILADELPHIA, SATURDAY, FEBRUARY 22, 1840. [Vol. III.

### A MEMOIR ON THE YELLOW FEVER OF THE WEST INDIES,

*As it occurred in the year* 1838, *at St. Pierre, island of Martinique.* By E. Rufz, D.M.P., Adjunct Professor at the School of Medicine of Paris.

Translated for the Medical Examiner, from the French Manuscript.—[*Concluded.*]

### OTHER CAUSES.

I have seen no person taken sick after any excess of venereal indulgence. There is less of this species of dissipation in the colonies than there is generally thought to be: the passion can be easily gratified, and is but little excited by the imagination.

As for fear, I have seen no immediate bad consequences result from it. Undoubtedly, this emotion, carried to a certain degree, must have a fatal tendency; but, on the whole, I would rather see persons fear the disease, than brave it. It is, without doubt, difficult to remain free from fear, when one sees his friends and companions falling around him; sorrow being conjoined with fear, there results from the combination a sort of torpor of all the functions, especially of the digestive. In the course of the epidemic I have seen many persons in this condition, but of these comparatively few who passed from that state to that of true yellow fever. Most commonly, as I have already said, the disease showed itself suddenly, and without any premonitory symptoms. I will give you a remarkable example of fear.

M. S——, a merchant, although he had been travelling in the different colonies for twelve or fifteen years, in consequence of some remarkable deaths in the city, was, on the 10th January, seized with so great a degree of alarm, that he sent for me. I found nothing the matter with him, except a slight anorexia, and a great depression of spirits. I cheered him up, and prescribed rest, a light diet, and amusements. Towards evening he again sent for me, and assured me that I did not pay sufficient attention to his condition, and that he was more unwell than I thought him to be. In vain I added my exhortations to those of his whole family; he persisted, during the whole night, in cramming himself with chicken water, and sweating himself under several blankets. Towards 6 o'clock, on the morning of the 11th January, took place the earthquake, which caused so much calamity in this island. M. S—— arose, much frightened; and, from this moment, having forgotten the yellow fever, he dreaded nothing but the earthquake.

The injurious influence resulting from the use of spirituous liquors, cannot be more precisely pointed out. But we must attribute chiefly to this the great mortality amongst soldiers and sailors. MM. Ventieghem and Lucotte offered very striking examples of this. Temperance societies would produce greater benefits in the colonies than in any other climate; but rum being a source of revenue to the country, they will always prefer having the yellow fever. Such is man!

Finally, among other circumstances favourable to the development of the disease, I have noted exposure to the sun's rays, as well as exposure to a cool atmosphere after being heated. More than one patient, attacked by the fever, had, just before they fell sick, taken a warm bath, either for the sake of cleanliness, or as a precaution, and had afterwards suddenly exposed themselves to a current of air.

### TREATMENT.

When this epidemic of yellow fever first broke out, as I was about to treat, for the first time, this terrible disease, I immediately had recourse to books as my first advisers. I soon found out that it was with the yellow fever, as with all the important diseases that afflict mankind: every possible mode of treatment had been tried, and they had all found supporters. However, through all the confusion of diversified testimony, I perceived, as I thought, that the antiphlogistic treatment pursued from the very commencement of the attack, was the plan which had the greatest number of advocates. Moreover, as the epidemic had first broken out at the military hospital, by attending to what was going on there, I learned that M. Catel, a strict follower of Broussais, but a conscientious man, and an esteemed physician, applied, with an extreme degree of rigour, antiphlogistic remedies, such as repeated blood-letting, leeches and drinks, diet and rest,—and that the mortality under his treatment was much less than that of the preceding epidemics. (See table.)

These two arguments in its favour determined my choice, and I resolved to treat the yellow fever antiphlogistically.

My first trial was not successful; it was the case of M. Lucotte. (See the account of his case.) I was, however, restrained by his relations, who were very much opposed to bleeding. The patient himself entertained the same opinion: I therefore tried only one large bleeding, and an application of forty leeches.

In the following case, the result will be seen to have been quite different.

The Abbé Grandidier, a man of a *canonical*

constitution—that is to say, corpulent and lymphatic, but nevertheless robust—felt some uneasiness during the day of 28th December: on the evening after, having assisted in the distribution of prizes at some institution, he was taken with a very severe chill, which was followed by heat. I saw him the next morning, (the 29th,) with the following symptoms: intense cephalalgia; injected conjunctivæ; dilated pupils; flushed face; general soreness of the muscles; pains in the loins; warm and moist skin; pulse one hundred and six, regular, soft and compressible; no nausea; no evacuation from the bowels; urine scanty and high-coloured; a severe and incessant cough, without expectoration; no rhonchus.

(A bleeding of twenty ounces, followed by syncope.)

In consequence of the bleeding, the cough became less troublesome, and the cephalalgia less severe; these symptoms, however, continued.

(At 3 o'clock in the afternoon, another bleeding of twenty ounces.)

30*th.*—He passed the night more quietly than the preceding. Face still flushed; cephalalgia continues; tongue whitish in the centre, without any redness; some nausea; abdomen yielding, and free from pain; one stool. The cough still persists; it is dry and harrassing, but auscultation gives no rhonchus, not even the mucous. Pulse soft, and one hundred; skin moderately warm. (Bleeding, sixteen ounces.) The same day, at noon, forty leeches were applied to the epigastrium.

On the 31st, the cephalalgia had ceased, but the cough still persisted, and was fatiguing to the patient. Pulse one hundred and six. (Thirty leeches to the epigastrium.)

On the 4th January, sixty leeches were applied behind the ears, and to the epigastrium, because he was still feverish, and had some cough.

On the eighth day the Abbé Grandidier was convalescent.

I have given a short account of this case, to show how far bloodletting may be carried. No one was more disposed to take the disease than this abbé, on account of—1st, his short residence in the island, having been here but ten months; 2d, his constitution, which was lymphatico-sanguine; 3d, his great dread of the disease; and, 4th, also on account of the particular epoch of the epidemic, it being most fatal about that time.

This case affords an example of that dry cough, almost entirely confined to the throat, which exists in some cases of yellow fever. I might report other similar cases; but to avoid superfluous details, which most men do not like to read, I will merely give the general results of my practice.

Of fourteen patients, whom I was enabled to bleed in the first twenty-four hours, two only died.

One of these was M. Lucotte. I think that if the bleeding could have been repeated in his case, the result would have been more favourable.

The other case is that of M. Ventieghem. Although antiphlogistic remedies were employed rigorously enough, (see the reflections accompanying the details of his case,) yet his circumstances were too unfavourable for any benefit to result from this treatment.

In two cases in which bloodletting was not commenced until forty-eight hours had elapsed, death took place. It is true, that in these cases, the treatment was very irregular, on account of the want of attendants.

The opinion of M. Catel, based upon a great number of facts, establishes a great difference between the cases in which bloodletting is resorted to in the first twenty-four hours, and those in which it is not tried until a later period. After forty-eight hours have elapsed, this physician prescribed leeches only; and he thinks that more benefit is to be expected from medicine.

He very freely communicated to me the following table of the mortality in the hospital, from the day on which bloodletting was commenced:

| | No. of patients. | Deaths. | Proportion of deaths to recoveries. |
|---|---|---|---|
| Patients treated during the first 24 hours, | 176 | 5 | 1 to 35 |
| Do. do. on the 2d day, | 108 | 11 | 1 to 9 |
| Do. do. after 3d day, | 143 | 40 | 1 to 3 |

Forty-two seamen, of the corvette Thisbé, being attacked with the yellow fever, were sent to the hospital, by M. Delussay, surgeon of the ship, as soon as they were taken sick; not one of these men died.

Here are two observations taken from my own practice, which prove, that even at an advanced period of the disease, bloodletting may produce very good results.

M. Dupouy, a man of a robust constitution, had reached the sixth day of an attack of yellow fever. He had been moderately bled at its commencement. I was called in consultation. His face was very red, his eyes injected, with tendency to turn upward; there was a marked stupour, slight delirium, no vomiting and no discharge from the bowels; suppression of urine for twenty-four hours; full pulse. We agreed to apply forty leeches behind his ears; three hours after, forty more were applied to the anus. The next day the patient was much better, but as he expectorated a little blood, twenty leeches more were put on his neck. The expectoraton ceased, and M. Dupouy got well.

M. B——, of a very strong constitution, had been bled by me at the commencement of his attack. On the third day, I had applied forty leeches to his epigastrium; as they bled for more than twenty-four hours, I arrested the bleeding. On the fourth day, when he seemed to be getting much better, he was taken with a very copious vomiting of blood, and threw up enough to fill a wash-hand basin, I did not hesitate to take sixteen ounces of blood from him. M. B. recovered entirely.

I am then of the opinion, 1st, that the employment of copious bloodlettings, from the very beginning of the yellow fever is one of the most efficacious means of combatting the disease.

2d. That even at an advanced period, it is still preferable to every other means, although more caution is necessary in its application.

But bleeding has not appeared to me to be useful as a prophylactic means. In the case of two persons whom I had occasion to bleed, although they were but slightly affected, yet it did not prevent the disease from declaring itself fifteen or twenty days after. I did not, indeed, try it on persons who were perfectly well.

In conjunction with bleeding, I ordered mild drinks, such as chicken water, weak lemonade, *orangeade*, emollient injections, repose, and diet.

Sometimes I confined myself to these last means only, without bloodletting.

I treated in this way eighteen persons, of whom not one died. It is true, that in these cases, the symptoms appeared to be less severe. But in three of these cases, the disease had commenced sharply, and I had only been prevented from employing venesection by the temperament of the patient not appearing to me to be sanguine enough.

*Purgatives.*—It is a custom of the country to give from the commencement of the disease, lemonade, and injections of cassia; this is the treatment of the mulatto women. In two cases, the treatment of which I undertook after this course had been commenced, and in which I let it continue in order to see its effects, I had cause to repent of it, for the patients died.

There was in the city a man of incredible ignorance as regards pathology, who gave medicines at random. This man having found, in M. Trousseau's Journal, a note by M. Piedagnel, on the good effects of purgatives in the treatment of *typhoid* fever, had it printed in the newspapers of the country, and began to apply it, without distinction, in all cases. Wishing at least to profit by his ignorance, as an additional test of the science, I directed my attention to this man's practice. He of all the physycians lost by far the greatest number of patients.

I do not, on this account, mean to say that purgatives are not to be used in yellow fever. I think that, in the early stage of the disease, after bloodletting, laxative injections, by emptying the intestines of fœcal matter, cannot but produce a beneficial effect.

When, towards the fourth or fifth day, there is obstinate constipation, with only a slight fever, and no vomiting, I have obtained good results from the use of a laxative of oil or cassia: but even in this case, if things are going on well enough, my advice is to wait, for the least disturbance may prove fatal. There are, however, two grave cases in which I derived much benefit from the administration of purgatives. One patient had had very bad stools of black blood: the administration of castor oil produced beneficial effects by freeing the intestine of this matter, (see case of Eliza.)

M. D——, on the ninth day of his sickness, after a very evident amelioration, was taken with vomiting, and threw up over two spoonfuls of black matter, similar to that of the black vomit. Thinking that it was the remains of an effusion of this matter, which had fortunately been arrested and had stagnated in the stomach, I directed him to take three spoonfuls of castor oil; he had three evacuations and threw up no more of this kind of matter.

From purgatives employed in extreme cases either to arrest the black vomit, or to combat the stupor occurring in the last moments, I have never seen any good results, when other means had failed, and when I had given it as my opinion that the patient would die.

*Of Emetics.*—The quack of whom I have spoken, at the commencement of the epidemic employed emetics in the first stage of the disease. As he had almost entirely given them up towards its end, I suppose that his want of success had been constant enough to oblige him to beware of them. Moreover, this remedy is rejected by almost all who have written on yellow fever. Reason, which, in default of experience, should guide us in the choice of therapeutic means, seems to repudiate emetics. Why, indeed, should we produce shocks of this kind on a man who has already a flushed face, red eyes, and intolerable pain in the head? why excite the appearance of a symptom which sometimes becomes too embarrassing?

One cannot say precisely what can be the use of frictions of lemon juice. It is a remedy which is very much boasted of, and is almost always employed simultaneously with others which are more active, such as bleeding or purgatives. But frictions require a degree of attention which we cannot expect from hirelings, to whom strangers attacked with the yellow fever are entrusted. It is rare that they are used with care for several successive hours. A service must not be very troublesome, if we would wish it to be performed accurately. I therefore lay little stress upon frictions; where I order them, I trust only to the exertions which are prompted by deep attachment.

As to the sulphate of quinine, and cinchona, I have made little use of them, and in the small number of cases in which I have tried them the results seemed equally balanced.

I confess that I should not venture, as I might have done, to give the cinchona at the beginning, when the patient is flushed, heated, in a state of apoplectic coma.

In two cases I have employed it without inconvenience after bloodletting, when the symptoms were already improving; so that I am not sure that the patients would not have got well without it.

In some children in whom the diseases seemed to assume a paroxysmal character, the sulphate of quinine was useful.

But, in the second period, when the patients got worse, whether they had been bled or not, I have three or four times used the sulphate of quinine without advantage. One of these patients had furious delirium, which was rare in this epidemic.

A physician in the town administered the quinine very largely. I was often called in consultation by him, and believe that he was less successful than those who trusted to antiphlogistics.

In conclusion, the administration of cinchona and of the sulphate of quinine, seems to me of doubtful benefit: from what I have seen, I should distrust them.

It remains for me to speak of blisters, which were employed in the most severe cases, conjointly with other means; it is difficult to appreciate their precise benefit. Their employment is often followed by sloughs, which are difficult of cure.

### CONCLUSIONS.

Such is the history of the epidemic which has desolated Martinique. I do not hesitate to regard it as one of the varieties of yellow fever:

1st. Because I find great similarity between my cases and those detailed by physicians who have observed previous epidemics of this disease.

2d. This epidemic reigned in some neighbouring colonies before reaching Martinique, but nothing shows that it was imported.

3d. This epidemic has been less fatal than other previous epidemics.

4th. We could detect no physical circumstance to which the origin of the disease could be attributed.

5th. Several of the most severe symptoms of yellow fever were rare in this epidemic, (chiefly sudden deaths, hæmorrhages, and severe nervous symptoms.)

6th. This epidemic was also remarkable from its attacking a considerable number of acclimated individuals, and even persons born in the country, especially children.*

7th. Large bloodletting employed at the beginning, seemed the most favourable treatment in Europeans.

---

*This probably arose from the long exemption of the island from yellow fever; hence acclimation, in a degree, ceased, and the inhabitants were placed nearly in the condition of the citizens of the Northern cities of America, in which yellow fever has from time to time prevailed.—Eds.

